# Therapeutic Inertia in Lipid-Lowering Treatment: A Narrative Review

**DOI:** 10.3390/jcm15031075

**Published:** 2026-01-29

**Authors:** Marco Vatri, Andrea Faggiano, Elisabetta Angelino, Marco Ambrosetti, Pompilio Massimo Faggiano, Francesco Fattirolli

**Affiliations:** 1 Dipartimento di Medicina Sperimentale e Clinica, Università di Firenze, 50121 Firenze, Italy; marco.vatri@gmail.com (M.V.); francesco.fattirolli@unifi.it (F.F.); 2 Dipartimento Cardio-Toracico-Vascolare, IRCCS Fondazione Ca’ Granda Ospedale Maggiore Policlinico, 20122 Milano, Italy; andreafaggiano95@gmail.com; 3 Laboratorio dell’Esercizio e dei Segnali Cardiorespiratori, Istituti Clinici Scientifici Maugeri IRCCS, 28013 Veruno, Italy; elisabetta.angelino@icsmaugeri.it; 4 U.O.C. Riabilitazione Cardiologica, ASST di Crema, Ospedale Santa Maria, 26027 Rivolta d’Adda, Italy; marco.ambrosetti@asst-crema.it; 5 Unità di Cardiologia, Dipartimento Cardiovascolare, Fondazione Poliambulanza Brescia, 25121 Brescia, Italy

**Keywords:** therapeutic inertia, lipid-lowering therapy, cardiovascular prevention, clinical phenotype, secondary prevention, implementation science

## Abstract

Therapeutic inertia in lipid-lowering treatment remains a striking paradox of modern cardiovascular medicine: at a time when the causal role of LDL-cholesterol in atherosclerotic disease is unequivocal and potent therapies are widely available, a substantial proportion of high- and very-high-risk patients still fail to receive timely treatment intensification. Contemporary European and international data consistently show fewer than one in three patients in secondary prevention achieve guideline-recommended LDL-C targets, revealing a persistent and unacceptable gap between scientific evidence and clinical reality. This narrative review examines therapeutic inertia as a key explanatory framework for this gap, describing its epidemiology, mechanisms, and clinical consequences in secondary cardiovascular prevention. We summarize the main physician-, patient-, and system-level determinants and propose recurrent clinician “phenotypes” of inertia that may help explain why opportunities are missed even in the highest-risk patients. The consequences are profound: therapeutic inertia contributes to what we propose as the conceptual framework of an “avoidable atherosclerotic burden”, the cumulative vascular injury that accrues each period in which LDL-C remains above target, translating into higher rates of avoidable cardiovascular events, and increased healthcare costs. Emerging strategies such as upfront combination therapy, decision-support systems, structured lipid pathways, and the integration of artificial intelligence offer practical tools to shift lipid management from reactive to proactive care. Overcoming therapeutic inertia is therefore not merely a matter of improving process metrics, but a clinical and ethical imperative. Closing the gap between evidence and practice requires transforming optimal lipid management from an exception into a system-level default, ensuring that every patient receives the full benefit of therapies proven to save lives. This work proposes a novel characterization of clinician ‘phenotypes’ and the concept of ‘avoidable atherosclerotic burden’ as a framework to understand and address this gap.

## 1. Introduction

### 1.1. The Established Efficacy of Lipid-Lowering Therapy

The direct causal relationship between the reduction in low-density lipoprotein cholesterol (LDL-C) and the decrease in the risk of events related to atherosclerotic cardiovascular disease (ASCVD) has been demonstrated by many years of clinical research [1]. Large-scale analyses, such as those by the Cholesterol Treatment Trialists Collaboration, have quantified this effect, showing that for every 1 mmol/L (about 38.7 mg/dL) reduction in LDL-C achieved with statin therapy, a 21–22% reduction in the risk of major vascular events was observed [1]. Subsequent research has reinforced the “lower is better” concept, indicating that an aggressive reduction in LDL-C to very low levels is not only safe but is associated with progressively greater clinical benefits, with no apparent lower limit beyond which the benefit ceases [1]. New and powerful classes of drugs, such as proprotein convertase subtilisin/kexin type 9 (PCSK9) inhibitors and small interfering RNA (siRNA)-based therapies, have made it possible to achieve once-unthinkable LDL-C targets, also demonstrating the advantageous effects of earlier intervention in achieving the therapeutic target (earlier is better) [2]. Importantly, the clinical benefit of LDL-C reduction appears remarkably consistent across different lipid-lowering drug classes. While agents differ in mechanism of action and magnitude of LDL-C lowering, cardiovascular risk reduction is largely proportional to the achieved LDL-C decrease, supporting a class-independent relationship between LDL-C exposure and atherosclerotic risk. This established evidence reinforces that the persistent gap in LDL-C target attainment is less a matter of therapeutic uncertainty and more the consequence of implementation failures and delayed treatment intensification [3,4].

### 1.2. The Gap Between Evidence and Clinical Practice

Despite established evidence and the availability of extremely effective drugs, a high proportion of patients at high and very high cardiovascular risk do not reach the recommended LDL-C targets [5]. This gap between what is theoretically possible and what is actually achieved represents the central problem in current lipid therapy management, similar to what is observed in hypertension and diabetes, where failure to achieve therapeutic goals is the rule rather than the exception [6]. After the publication of the 2019 ESC/EAS guidelines, numerous studies (Table 1) have shown that only a minority of patients with ASCVD achieve the LDL-C targets. The multinational DA VINCI study reported that only about 18% of very high-risk patients had an LDL-C level < 55 mg/dL [7]. Similar results were observed in the SANTORINI study, in which about 20–22% of patients at high or very high cardiovascular risk had reached the target despite the widespread use of statins [8]. The INTERASPIRE study extended these observations worldwide, showing that only 16.6% of patients with coronary artery disease reached an LDL-C level < 55 mg/dL one year after hospitalization, with a marked gender disparity (12.0% in women vs. 17.9% in men) [9]. Beyond gender, significant racial disparities also exist; evidence suggests that ethnic minorities often receive less intensive treatment and lower statin prescription rates, partly driven by lower risk perception and communication gaps between clinicians and patients [10]. Finally, the Italian BRING-UP Prevention registry reported that only 33% of patients in secondary prevention had reached target LDL-C levels, despite an increase in statin and ezetimibe prescriptions, while PCSK9i and inclisiran remained underutilized [11]. Complementing these results, the ITACARE-P registry offered a detailed picture of lipid management in Italian patients with ASCVD referred to structured cardiovascular rehabilitation and secondary prevention programs. In this cohort, 43% of very high-risk patients reached an LDL-C level < 55 mg/dL, while only 18% of those at extreme risk reached an LDL-C level < 40 mg/dL. Among patients who did not reach the target, lipid-lowering therapy (LLT) remained unchanged after a follow-up visit in 51% of cases, and even among those whose treatment was modified/intensified, only 42% received an adjustment hypothetically sufficient to reach the LDL-C targets recommended by the guidelines [12]. These observational studies provide a valuable picture of real-world lipid management but include limitations that likely lead to an underestimation of the true burden of inertia. Patients are often recruited in structured centers (selection bias toward better care and adherence), pharmacological titration details and timing are frequently incomplete, and the heterogeneity of health systems affects access to innovative therapies. Furthermore, many analyses classify intensification dichotomously (yes/no) without capturing contextual drivers such as comorbidities, contraindications, or patient preferences. Overall, the persistence of low target attainment despite increasingly effective therapies indicates that the problem is less a lack of evidence or tools and more a failure of implementation. Additional details on study design, populations, limitation and target attainment rates across contemporary registries are summarized in Table 1.

This underuse of treatment generates a paradox: while our knowledge expands, targets become more ambitious, and drugs more potent, the gap between evidence and clinical practice does not narrow but widens. This suggests that the problem lies not in a lack of scientific guidance or therapeutic tools, but in a failure of optimal implementation. Simply generating new evidence or developing more effective drugs proves to be an insufficient strategy if the barriers limiting their application in clinical practice are not addressed.

### 1.3. Therapeutic Inertia as a Primary Cause

Therapeutic inertia can be broadly defined as the failure to initiate or intensify treatment when clinical goals are not met, despite clear indications to do so [6]. Although some instances of non-intensification may be clinically justified (e.g., competing comorbidities, contraindications, limited life expectancy, or patient preferences), a large proportion of missed opportunities in lipid management appears attributable to modifiable inertia [15]. In this narrative review, we summarize the epidemiology of therapeutic inertia in lipid-lowering therapy, discuss its main mechanisms and determinants, describe its potential clinical consequences, and highlight practical solutions to overcome inertia in routine care.

## 2. Defining Inertia: Terminology

**Figure 1:**
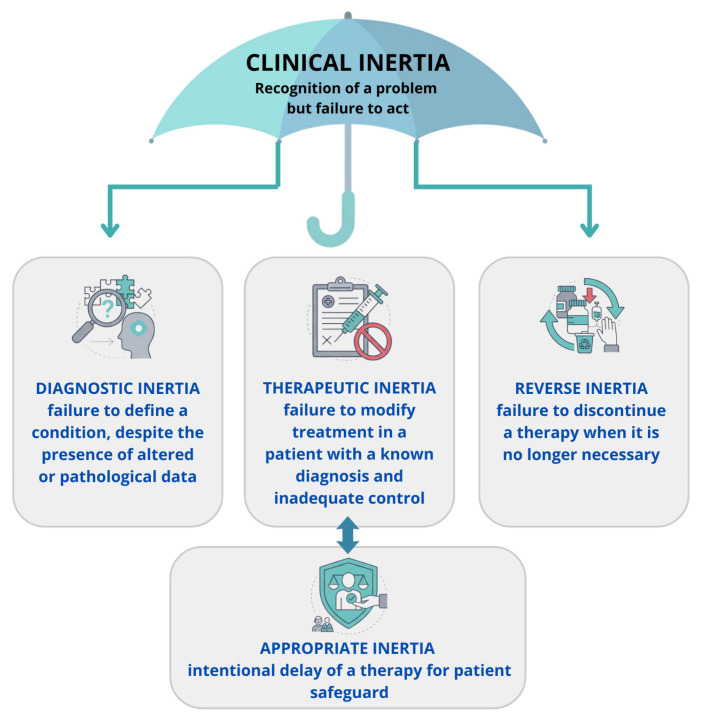
Terminology of inertia.

Terminological precision is not merely semantic, it directly affects how therapeutic inertia is measured, reported, and ultimately addressed. In research, different definitions can lead to heterogeneous estimates of inertia and make comparisons across studies problematic. In clinical practice, distinguishing true therapeutic inertia from “appropriate” non-intensification is essential to avoid both undertreatment and overtreatment, and to ensure that implementation strategies target modifiable barriers rather than justified clinical decisions. See Figure 1.

- Clinical inertia was introduced in the literature in 2001 to describe a behaviour defined as “recognition of the problem, but failure to act” [16]. This term applied mainly to the management of risk factors, such as hypertension, diabetes, or dyslipidemia, in contexts where therapeutic goals, the benefits of achieving them, and available therapies were clearly defined. In subsequent years, the need for more precise terminology emerged to distinguish different forms of inertia, considering clinical inertia an “umbrella” term that includes screening, diagnosis, management of non-pharmacological risk factors, and referral to other specialist sectors [17].
- Therapeutic inertia is considered a specific component of clinical inertia, relating to the failure to modify treatment in a patient with a known diagnosis and inadequate control, differentiated from “diagnostic inertia” considered as the failure to define a condition, despite the presence of altered or pathological data [18].
- Appropriate inertia describes clinically justified non-intensification, for example due to contraindications, documented intolerance, competing priorities, limited life expectancy, or informed patient preferences. This distinction is essential to avoid conflating high-quality individualized care with suboptimal or unjustified undertreatment [19].
- Reverse inertia refers to failure to de-intensify or discontinue therapy when it is no longer indicated or when risks outweigh benefits (e.g., persistent adverse effects, contraindications, or changes in overall risk–benefit profile). Although less discussed in lipid management, it highlights that inertia can also occur in the direction of overtreatment [18,19].

## 3. Therapeutic Inertia in Secondary Prevention

For most authors, therapeutic inertia refers more specifically to the failure to intensify drug therapy. Translating this abstract concept into a measurable metric is fundamental for research and for monitoring the quality of care. Clinical and epidemiological studies related to LLT have described therapeutic inertia in various ways, depending on the clinical context.

- Secondary prevention post-PCI: the failure to prescribe a high-intensity statin within 30 days of discharge after a percutaneous coronary intervention is a clear metric of inertia, given that guidelines universally recommend this approach [20].
- Chronic management: the observation that drug treatment remains unchanged during a follow-up visit, despite LDL-C levels being above the recommended target for that risk category (e.g., >55 mg/dL in a patient with established ASCVD), constitutes the most common definition of inertia in outpatient practice [21].
- Combination therapy: The failure to add a non-statin agent (such as ezetimibe, bempedoic acid, or a PCSK9 inhibitor) in a patient who, despite taking the maximum tolerated statin dose, does not reach the LDL-C target, represents a more advanced but equally critical form of inertia [22].

To illustrate how therapeutic inertia may manifest in routine clinical practice and how suboptimal intensification strategies can delay LDL-C target achievement, we present a representative clinical vignette (Box 1).

Box 1 **Clinical Vignette: Missed Opportunity for Combination Therapy.**

A 56-year-old patient with recent Acute Coronary Syndrome (ACS) was discharged on rosuvastatin 20 mg/day. At the 8-week follow-up, LDL-C was 68 mg/dL (23% above the recommended target). The physician opted to double the rosuvastatin dose (to 40 mg/day).

*Analysis:* Based on the “rule of 6,” doubling the statin dose provides an additional reduction of approximately 6%. Consequently, the patient is unlikely to reach the therapeutic goal. This scenario exemplifies therapeutic inertia, characterized here by suboptimal intensification. A more evidence-based approach would have been the addition of Ezetimibe, which offers an expected further reduction of approximately 25%

From an implementation perspective, it is important to distinguish how therapeutic inertia is defined in different settings. In quality improvement programs and audits, inertia is often operationalized through simple binary indicators, such as the absence of treatment intensification within a predefined timeframe (e.g., 4–12 weeks after an LDL-C value above target) or failure to prescribe high-intensity statin therapy at discharge after an ASCVD event. In contrast, research studies typically adopt more nuanced longitudinal definitions, incorporating repeated LDL-C measurements, treatment trajectories, dose optimization, treatment adherence, access constraints, and clinical justifications for non-intensification (“appropriate inertia”).

Therapeutic inertia is intrinsically linked to the evolution of guidelines [23] with a complex dynamic: as guidelines have introduced increasingly lower LDL-C targets for very high-risk patients, the potential “space” for inertia has expanded. Every new, more aggressive recommendation creates a new cohort of patients who, overnight, find themselves “not at target”. Consequently, the measured prevalence of inertia can paradoxically increase immediately after the publication of new guidelines, because the goals have become more ambitious. This implies that the simple dissemination of new guidelines must be accompanied by implementation support programs; otherwise, the guidelines themselves, despite being well-formulated, risk widening the gap they aim to close. Therefore, contemporary quality indicators should not only capture goal attainment but also the timeliness and appropriateness of treatment intensification relative to the prevailing guideline standards.

### Quantifying the Prevalence

Therapeutic inertia is not an anecdotal problem or limited to specific contexts, but a widespread and quantifiable public health phenomenon, as demonstrated by a growing number of studies conducted in different populations and clinical settings. Epidemiological data paint a worrying picture, highlighting how “failure to act” is a common practice throughout the cardiovascular disease care pathway.

- Acute phase (post-PCI): In a national Korean study that analyzed data from over 204,000 patients undergoing percutaneous coronary intervention, therapeutic inertia, defined as the failure to prescribe a high-intensity statin, was identified in 64.1% of cases. This figure is particularly alarming, as this is a very high-risk population where the indication for aggressive therapy is unequivocal [20].
- Management of chronic ischemic heart disease: A multicenter Spanish study examined outpatient practices for patients with ischemic heart disease and dyslipidemia, finding therapeutic inertia in 43% of medical visits. In these cases, despite LDL-C being above target, no modification was made to the therapy [5].
- Secondary prevention in Italy: In secondary prevention, among 1074 patients not at LDL-C target, no change in lipid-lowering therapy was proposed in 51.4% of cases. In practice, for more than one in two patients, the physician chose not to act [24].
- Patients with diabetes mellitus: Lipid management in diabetic patients, who are frequently at very high cardiovascular risk, is another critical point. A study conducted in a primary care setting found a prevalence of therapeutic inertia of 66.1% among patients with type 2 diabetes and suboptimal lipid control [25].
- General population with ASCVD: An analysis of a large US cohort highlighted a massive underutilization of recommended therapies. Only 39.4% of patients with ASCVD were on high-intensity statin therapy, while 23.9% were not taking any statin. The use of non-statin therapies was extremely low, with only 4.4% of patients on ezetimibe [24].

In summary, available evidence suggests that therapeutic inertia affects roughly 30–50% of patients in secondary prevention, depending on the operational definition and setting.

Paradoxically and counterintuitively, patients with the lowest LDL-C targets (e.g., ≤40 mg/dL) were less likely to have their therapy intensified. This phenomenon suggests a kind of hesitation on the part of physicians when faced with goals perceived as too difficult to achieve. This epidemiological data reveals a profoundly worrying phenomenon, which can be defined as a “Risk-Treatment Paradox”. The highest-risk populations who would derive the greatest absolute benefit from aggressive LLT, are those in which there is an inverse relationship between the need for treatment intensification and the likelihood that intensification is actually occurring. This represents a critical failure point in the translation of scientific evidence from the guidelines to the individual patient.

Notably, the risk–treatment paradox observed in lipid management is not unique. Similar paradoxes have been described across cardiovascular prevention, including suboptimal use of evidence-based therapies in patients at the highest absolute risk (e.g., intensive blood pressure control, heart failure therapies, and anticoagulation strategies), reflecting common implementation barriers and clinician decision-making biases. Recognizing this recurring pattern strengthens the case for addressing therapeutic inertia as an implementation science priority.

## 4. Mechanism of Inertia

Therapeutic inertia is not the result of a single factor, but a complex phenomenon that emerges from the dynamic interaction of barriers at the physician, patient, and healthcare system levels. These domains should not be interpreted as mutually exclusive, as they frequently overlap and reinforce each other in clinical practice. Understanding this multifactorial etiology is the prerequisite for designing effective interventions. A tripartite model offers a useful framework for analysing and classifying the different causes. This framework is summarized schematically in central illustration, while the main determinants are detailed throughout this section and in the accompanying tables.

### 4.1. Physician-Related Factors

The roots of inertia at the physician level are deep and complex, going beyond a simple lack of knowledge. For example in primary care, inertia is often driven by workload, competing demands and lower familiarity with advanced LDL-C lowering pathways [16]. In specialist settings, inertia may more frequently reflect overconfidence, fragmented care transitions, or inappropriate reassurance by partial LDL-C improvement despite unmet targets [26].

#### 4.1.1. Cognitive and Psychological Barriers

The physician’s decision-making process is influenced by a series of cognitive biases and psychological factors. One of the most insidious barriers is the overestimation of the quality of care. Physicians often perceive their patients to be better controlled than objective data indicates, which leads to a false sense of security and a reduced perception of the need to act [27]. This is compounded by a reluctance to change the status quo. Changing a therapeutic regimen that a patient has been on for a long time and which is apparently well-tolerated, is perceived as an action that carries risk (of adverse effects, of non-adherence), while not acting is seen as the safer choice [28]. This tendency manifests in “repetitive prescribing”, where the previous therapy is renewed uncritically without re-evaluation based on the latest analyses and guidelines [20]. Fear of adverse effects, particularly muscle-related ones associated with statins, is another powerful driver of inertia. This concern, is often disproportionate to the real risk and leads physicians to prescribe a low initial dose and hesitate to increase it, even when the patient reports no symptoms [20]. Finally, physicians may resort to “soft excuses” to justify inaction, such as attributing the lack of control to the patient’s poor adherence or deciding to “wait and see” at the next visit, thereby postponing a necessary decision [29].

#### 4.1.2. Knowledge and Training

Although not the only factor, the lack of up-to-date knowledge plays a significant role. Lipid management guidelines are constantly evolving, and keeping up with new targets, new drugs, and complex treatment algorithms can be challenging [6]. Despite the causal, genetic, and pharmacological demonstration of the role of LDL-C in the progression of atherosclerotic plaque, several studies show a persistent clinical underestimation of its pathogenetic weight. As highlighted in recent reviews, cholesterol is often perceived as one among many risk factors, rather than as the primary driver of the atherosclerotic process, resulting in a failure to intensify LLT even in high-risk patients [30]. Studies have shown that specific training, for example in family medicine, is associated with less inertia, suggesting that targeted educational programs can improve clinical practice [25].

## 5. Phenotypes of Inertia with Clinical Responsibility

The complex interaction between the described factors has been addressed from different perspectives, but with a prevailing focus on analyzing what is attributable to the patient and the healthcare system. As reported in the most recent literature, physicians’ responsibility makes it necessary to identify different profiles in order to outline the most recurrent “phenotypes”. These phenotypes are not mutually exclusive and may overlap within the same clinician depending on context (e.g., workload, setting, and familiarity with therapies), and may shift over time.

- “Unaware/Uninformed”: This phenotype is characterized by gaps in knowledge of new available therapeutic options, or by the failure to recognize a patient who is not at target due to inadequate monitoring. The causes may be insufficient professional development or, even more so, information overload that prevents the assimilation of relevant clinical updates [31,32]. Corrective strategies: focused education, guideline simplification, audit/feedback, and decision-support tools to prompt timely intensification.
- “Aware but Hesitant”: Perhaps the most common phenotype. There is awareness of the clinical problem and theoretical knowledge of the solutions, but direct experience with certain therapies is lacking. The introduction of new drug classes, with different mechanisms of action and safety profiles, generates uncertainty, feeding a vicious cycle where non-use prevents the accumulation of experience, and the lack of experience fuels hesitation, preferring to stick to more familiar, albeit less effective, drugs [31,32]. Corrective strategies: mentoring, shared decision-making tools, streamlined reimbursement pathways, and safety-focused messaging to improve confidence in intensification.
- “Acquired Certainties”: The profile is characterized by resistance to change and a preference for established routines; low conviction of the advantages of new drugs, lack of mental automaticity in their use, and difficulty in communicating effectively with the patient to explain and convince them of the need for the cure or a change [31,32]. Corrective strategies: behavioral nudges, peer benchmarking, case-based discussion, and multidisciplinary pathways to counter entrenched routines.
- “Overwhelmed/in Burnout”: Inertia here is not primarily a choice or a knowledge gap, but a symptom of stress and systemic pressure. The clinician is in a hurry, demotivated, and has exhausted the mental resources to engage in the decision-making process necessary for therapeutic intensification. They may tend to choose the least demanding options and avoid the complex cognitive and emotional effort of renegotiating therapy with the patient [31,32]. Corrective strategies: workflow redesign, nurse/pharmacist-led titration clinics, automated pre-visit planning, and reduction in administrative burden.

Table 2 details the specific cognitive mechanisms (biases) that often underpin the clinical phenotypes described above.

## 6. Patient-Related Factors and the Challenge of Non-Adherence

The patient is not a passive recipient of care but a central actor whose behavior and beliefs profoundly influence therapeutic decisions:

- Poor adherence to therapy is perhaps the most critical patient-level factor, with statin discontinuation rates reaching almost 60% at one year [28]. Adherence and inertia are trapped in a vicious cycle: a physician may be reluctant to intensify a therapy if they suspect the patient is not taking it correctly, while patients may stop taking a drug they perceive as ineffective (because the physician never optimized it) or that they fear may cause harm [35].
- The patient’s beliefs and perceptions are decisive. The direct visualization of atherosclerotic plaque reduces clinical inertia because it makes the cardiovascular risk “tangible” for both the physician and the patient. In the VIPVIZA trial, the simple visual communication of carotid plaques led to a significant reduction in estimated risk and an increase in the use of statins and lifestyle interventions compared to standard care [36]. Similarly, the awareness of positive coronary artery calcium (CAC) findings has been shown to significantly improve lipid-lowering medication adherence, as it provides tangible evidence of risk [37]. Conversely, the perceived risk of adverse effects is often exaggerated, fueled by anecdotal information or information from unreliable sources.
- Differences in perspective between physician predictions and patient’s perceptions. Recent study reports that physicians overestimated patient satisfaction with drug information: for example, believing that 75% of patients were satisfied with information on side effects, while only 51% of patients were. The misalignment also manifested in treatment goals: 72% of patients expressed at least one doubt about their personal need to take the therapy, only 36% believe that LLT are vital for their current and future health, and only 50% stated they were satisfied with the information on side effects [38].
- Socioeconomic factors, such as the cost of medications, and demographic factors, further contribute to creating barriers to accessing and maintaining therapy [24]. Cultural and health-literacy factors should also be considered important modifiers of adherence and therapeutic inertia. Limited health literacy, language barriers, culturally mediated beliefs about chronic preventive therapies, and varying levels of trust in healthcare systems may influence risk perception and willingness to initiate or maintain long-term LLT. These factors are particularly relevant in multicultural settings and may contribute to disparities in LDL-C goal attainment.

## 7. Structural and Healthcare System Determinants

The decisions of physicians and patients take place within a context, the healthcare system of the country in which they live, which can facilitate or hinder action.

- The clinical work environment is often characterized by strong time pressure. Short and rushed visits do not allow for an adequate review of data, effective patient education, and a shared decision-making process, all of which are essential elements for overcoming barriers to therapy intensification [6].
- The complexity of guidelines, while an indispensable tool, can become a barrier when their application requires time and cognitive resources that are not available during a routine visit [27].
- Structural barriers such as limited access to specialist care and drug costs play a crucial role. The high costs of the most innovative drugs, such as PCSK9 inhibitors, can limit their prescription due to healthcare system budget constraints or unsustainable costs for the patient [28].
- Local regulatory systems often impose contradictory reimbursement restrictions that limit access to high-potency drugs despite guideline recommendations. Fragmentation of care, with poor communication between general practitioners and specialists, can lead to a diffusion of responsibility, where no physician feels fully responsible for the long-term management of the patient’s lipid profile [39].

Addressing these determinants requires a two-tiered approach. On one hand, structural barriers like reimbursement criteria and costs demand long-term policy advocacy. On the other, process-level barriers such as fragmented communication and time constraints can be mitigated in the short term through organizational redesign and the adoption of more efficient care pathways.

The traditional view of inertia as an exclusively “physician” problem is a harmful simplification. The data strongly supports a model of reciprocal inertia, in which factors at the physician, patient, and system levels create a self-reinforcing negative feedback loop that paralyzes clinical action. Inaction, therefore, is not a single failure, but a multi-level condition that requires interventions capable of acting simultaneously on multiple points in this chain.

Table 3 summarizes the determinants of inertia attributable to clinician, patient and healthcare system.

## 8. Clinical Consequences of Inertia

The link between inertia and worsening clinical outcomes has been quantified alarmingly. A landmark study showed that, in high-risk dyslipidemic patients, the presence of therapeutic inertia is associated with a 2.18-fold increase in the risk of a first ischemic event (such as myocardial infarction or stroke) within just 18 months [41]. The mechanism underlying this increased risk is the accumulation of exposure to high LDL-C levels. The time a patient remains above their lipid target due to inertia contributes to the progression of atherosclerotic plaque with long-term consequences, as a greater risk of stent or bypass occlusion, re-infarction, the need for new revascularization procedures, and an increased risk of death [1].

To conceptualize the cumulative damage caused by inertia, it is useful to borrow and adapt the concept of “avoidable glycemic burden” described in the context of diabetes [19]. We propose the theoretical framework of an “avoidable atherosclerotic burden”, which represents the time-integrated exposure to LDL-C levels above the target that could have been prevented with timely therapeutic action. Prolonged exposure to a pro-atherogenic environment induces persistent epigenetic and inflammatory changes in the endothelium, which may not be completely reversible even when lipid control is later achieved [30]. The clinical impact of therapeutic inertia is not linear, but is likely exponential and “front-loaded”, i.e., concentrated in the initial phases. Based on the analogy with the “legacy effect” observed in diabetes, where early intensive glycemic control confers cardiovascular benefits decades later, it is possible to hypothesize a “lipid legacy effect”: a delay of just one year in reaching the LDL-C target after a first myocardial infarction could cause irreversible vascular damage. This consideration redefines inertia not just as a delay, but as the waste of the most critical window of opportunity for secondary prevention [36].

The traditional “start low, go slow” model is intrinsically vulnerable to inertia. An alternative paradigm, especially for very high-risk patients, is the now-established “upfront” combination therapy, starting immediately with a high-intensity statin plus ezetimibe. This approach allows the target to be reached more often and quickly, reduces the number of visits needed for titration, and, if available in a single-pill formulation, can significantly improve adherence [2]. In summary, the most powerful strategies to overcome inertia are those that create “systems of automaticity”, which avoid or support the fallible decision-making processes of physicians and patients. The implication is that interventions to combat inertia should be deployed with maximum aggressiveness and urgency immediately after a sentinel event (such as an infarction or PCI), as it is at this moment that the clinical return on investment of timely action is greatest.

## 9. Multi-Dimensional Strategy to Overcome Therapeutic Inertia

Addressing such a complex and deep-rooted problem as therapeutic inertia requires abandoning simplistic solutions and adopting synergistic interventions that act simultaneously on the different levels of the problem: the system, the care team, the physician-patient dyad, and pharmacological therapies. Identifying inertia clinician phenotypes is only the first step. Just as happened in the past with patient adherence, the crucial question is whether and how these profiles might be more susceptible to change than others. There are no one-size-fits-all solutions to resolve inadequate therapeutic intervention: a credited approach is to identify the clinician’s phenotype to outline some hypotheses for support or correction. The key to success should lie in adopting a “precision” approach, mapping specific, evidence-based interventions targeted at the barriers that may characterize each clinician’s phenotype.

The structured setting of Cardiac Rehabilitation provides an ideal care environment for overcoming therapeutic inertia: the support of a multidisciplinary team combined with close clinical and biochemical monitoring makes it possible to dismantle some of the mechanisms underlying inertia and to safely and effectively implement therapeutic intensification [12]. Cardiac rehabilitation offers a structured post-ASCVD follow-up window to reassess LDL-C early and implement protocol-driven intensification. These elements (LDL-C checkpoints, standardized algorithms, and nurse-supported titration) can be replicated in routine outpatient care even outside dedicated CR centers.

## 10. Future Perspectives and Open Questions

Despite progress in understanding and combating therapeutic inertia, important areas of uncertainty and opportunities for future research and innovation remain. The goal is to move from reactive interventions to proactive and personalized strategies to prevent inertia from taking hold. Most studies on interventions against inertia have focused on process measures (e.g., prescription rates) or surrogate endpoints (e.g., LDL-C levels). There is a shortage of long-term data on the impact of different strategies on cardiovascular outcomes (morbidity and mortality) and their cost-effectiveness [42]. Demonstrating benefits not only on lab values but also on patients’ lives and system sustainability is the crucial next step to developing methodologies for large-scale adoption. Another critical area of research concerns health disparities. Although it has been identified that inertia has a greater impact on women, the elderly, and ethnic minorities, the effectiveness of interventions specifically targeted at reducing these disparities is still poorly studied [43]. Future research should move from descriptive registries to implementation-focused study designs that can test inertia-reducing interventions. Key endpoints should include not only LDL-C target attainment but also process metrics such as time-to-intensification, time-to-target, and proportion of eligible patients receiving combination therapy within a predefined window (e.g., 4–12 weeks post-event). Additional relevant outcomes include medication persistence/adherence, equity metrics (sex and ethnicity gaps in intensification), clinician-level performance indicators, and, when feasible, hard clinical outcomes (MACE) and cost-effectiveness.

## 11. Role of Artificial Intelligence and Predictive Analysis

The future of the fight against inertia will also lie in the use of more advanced technologies. Artificial intelligence (AI) and machine learning algorithms have large potential: by analyzing datasets from electronic health records, AI algorithms will be able to identify complex patterns and develop predictive models based on dozens of variables (including clinical history, adherence, sociodemographic profile, prescribing style), capable of generating an “inertia risk score”. Concrete examples include Electronic Health Record integrated alerts that flag high-risk patients directly during visits, which have shown success in increasing statin prescription rates compared to standard care [44]. This would allow targeted interventions to be activated, such as enrolment in a management and pharmacological guidance program or the sending of personalized alerts, before the patient accumulates months or years of suboptimal control [44]. Finally, the deployment of AI tools must address ethical concerns regarding data privacy and the risk of algorithmic bias, ensuring that automation supports rather than replaces clinical judgment.

## 12. Conclusions

Therapeutic inertia in the management of dyslipidemias represents one of the greatest contradictions of modern medicine. In an era of unprecedented scientific discoveries and extraordinarily effective therapeutic options, widespread and persistent inaction prevents millions of patients from receiving the benefits of decades of research. This phenomenon is not a simple process flaw, but a systemic failure in translating scientific evidence into clinical practice, with serious consequences in terms of morbidity, mortality, and costs to society. Our analysis has shown that inertia is a pervasive problem, rooted in a complex interaction of physician-, patient-, and healthcare system-related factors, which reinforce each other creating a vicious cycle. We have highlighted how this problem generates an unacceptable paradox where the highest risk is associated with the least aggressive treatment. The consequences of this failure produce a significant increase in the risk of recurrent ischemic events and the accumulation of an “avoidable atherosclerotic burden” that compromises the long-term health of patients. However, we are not powerless in the face of this challenge. There is a solid body of evidence supporting a multi-dimensional approach to overcoming inertia. This approach must synergistically integrate the redesign of care systems through technology and multidisciplinary teams, the strengthening of the physician-patient relationship through education and shared decision-making, and the intelligent adoption of new pharmacotherapeutic paradigms. The key to success lies in creating systems that make the correct action the easiest and most automatic choice, transforming adherence to guidelines from a conscious effort to a default process.

Overcoming therapeutic inertia is not simply a quality improvement goal but an ethical imperative, while acknowledging the systemic pressures clinicians face. It is a professional and moral duty to ensure that every patient receives the standard of care that scientific evidence has shown to be effective in saving lives and preventing disability. Closing the gap between evidence and practice is no longer an option, but an urgent necessity for global cardiovascular health.

Reducing therapeutic inertia should become a routine quality goal of secondary prevention pathways.

## Figures and Tables

**Table 1:** Clinical trials on hypercholesterolemia treatment in secondary prevention.

Trial	Sample Size (n)	LDL C Target (mg/dL)	% Achieved	Study Limitations	Reference
**Da Vinci**	2888 in secondary prevention	<70 * <55 **	39% 18%	- bias in the choice of therapy, from pre-treatment LDL-C levels and local prescribing restrictions. - “best-case” scenario, may not fully reflect general clinical practice”	[7]
**Santorini**	9044 at high or very high cardiovascular risk	<55 **	18.4%	- observational study did not examine medication adherence, lifestyle, or control of other risk factors - “best-case” scenario, may not fully reflect general clinical practice”	[8]
**Interaspire**	4548 after hospitalization for a coronary event	<55 **	16.6%	- conducted in heterogeneous geographical areas (very different healthcare practices) - initiated during the COVID-19 pandemic: patient selection bias	[9]
**Bring Up P.**	4790 patients with a previous atherothrombotic event	<55 **	33%		[11]
**Itacare**	1909 with previous atherosclerotic cardiovascular disease	<55 ** <40 **	43.6% 18.2%	- Lifestyle interventions not considered - Single LDL measurement does not account for possible fluctuations over time	[12]

* 2016 ESC/EAS guidelines for the management of dyslipidaemias [13]. ** 2019 ESC/EAS guidelines for the management of dyslipidaemias [14].

**Table 2:** Mechanisms underlying physician inertia.

Cognitive Mechanism	Action	When It Occurs	Effects in Clinical Practice	Reference
**Omission**	Preferring inaction over action	Fear of making a wrong choice	Distorted application of primum non nocere	[33]
**Anchoring**	Not updating one’s judgment in light of new clinical data		Deciding by comparing only with a limited set of known and familiar elements remaining tied to the initial diagnosis or therapeutic plan	[26]
**Mental shortcut**	Judging an event as more likely just because it is easier to recall		Reaching conclusions quickly using intuitive and hasty mental processes, avoiding cognitive efforts. The recent management of a serious side effect of a therapy can make one overestimate the probability of it recurring.	[26]
**Confirmation bias**	Seeking and interpreting information in a way that confirms one’s pre-existing beliefs and prejudices	Managing to decide in complex and uncertain situations where change is frightening	Giving more weight to clinical data that support the chosen choice (not to intensify) and minimizing those that contradict it.	[26]
**Self-protection bias, or convenient excuses**	Shifting one’s decision-making responsibilities onto others, onto external factors		Protecting one’s self-esteem, one’s positive self-image. Attribution of the failure to intensify therapy to the patient’s poor adherence, with inadequate assessment of events and risk of perpetuating the same errors.	[15]
**Competing demands**	Prioritizing immediate and symptomatic problems	Managing to decide quickly in the face of a sense of time urgency	Asymptomatic and chronic conditions may not receive adequate attention; additional concerns reduce the likelihood of therapy modification by 49%.	[34]

**Table 3:** Causal Factors of Therapeutic Inertia.

Responsibility	Causal Factor/Barrier	Description and Mechanism	Reference
**Physician**	Overestimation of care quality	Physicians tend to believe their patients are better controlled than they actually are, failing to recognize the need to act.	[29]
	Reluctance to change established therapies	Hesitation to change long-standing, suboptimal, therapeutic regimens for fear of destabilizing a perceived balance.	[28]
	Fear of adverse effects	Concern about side effects associated with statins, especially at high doses, leads to not initiating or not titrating therapy.	[20]
	Soft excuses and clinical uncertainty	Using justifications (e.g., “the patient is non-adherent”) to postpone the decision; discomfort with diagnostic or therapeutic uncertainty.”	[6]
	Repetitive prescribing	Practice of renewing the previous prescription without a critical re-evaluation based on laboratory data and current guidelines.	[20]
	Training deficiencies	Lack of up-to-date knowledge on the latest guidelines, new drugs, or complex therapeutic strategies.	[25]
**Patient**	Poor adherence to therapy	Failure to take medication as prescribed, which can be interpreted by the physician as drug ineffectiveness, discouraging intensification.”	[28]
	Negative beliefs and perceptions	Fear of side effects, distrust in medications, perception that high cholesterol is not a “real” disease because it is asymptomatic.	[28]
	Poor knowledge of the disease	Lack of understanding of the chronic nature of dyslipidemia and the long-term benefits of therapy, which reduces motivation to follow treatment.”	[40]
	Socioeconomic factors	Costs of medications that represent a barrier to purchase and adherence.	[28]
	Comorbidity and polypharmacy	The management of multiple conditions and medications can lead to “therapeutic overload”, reducing the patient’s ability to adhere to an additional drug or a higher dosage.	[6]
**Healthcare System**	Time and organizational constraints	Visits that are too short, leaving insufficient time for in-depth discussion, patient education, and shared decision-making.	[6]
	Complexity of guidelines	Guidelines, while essential, can be long, complex, and difficult to apply in the limited time of a visit.	[6]
	Structural barriers	Limited access to specialist care; high costs of newer drugs that limit their prescribability by the system.	[28]
	Fragmentation of care	Lack of coordination and communication between general practitioners and specialists, which leads to a dilution of responsibility	[39]
	Lack of support systems	Absence of automatic recall systems or decision support integrated into electronic health records to flag patients not at target.	[1]

## Data Availability

No new data were created or analysed in this study. Data sharing is not applicable to this article.

## Abbreviations

The following abbreviations are used in this manuscript:

LLT	Lipid-Lowering Therapies
LDL-C	Low-Density Lipoprotein Cholesterol
PCI	Percutaneous Coronary Intervention
ASCVD	Atherosclerotic Cardiovascular Disease
PCSK9	Proprotein Convertase Subtilisin/Kexin type 9
siRNA	small interfering RNA
ESC/EAS	European Society of Cardiology/European Atherosclerosis Society
